# Liver myofibroblasts regulate the phenotype and function of monocytes through soluble factors in cirrhosis

**DOI:** 10.3892/etm.2012.767

**Published:** 2012-10-25

**Authors:** MIN ZHANG, FENG-LAN WANG, JIAN-YUN ZHU, YU-BAO ZHENG, QI-YI ZHAO, YU-RONG GU, QI ZHANG, YU-TIAN CHONG, ZHI-LIANG GAO

**Affiliations:** 1 Department of Infectious Diseases, The Third Affiliated Hospital, Sun Yat-sen University, Guangzhou;; 2 Department of Infectious Diseases, The First Affiliated Hospital of Medical College of Xi’an Jiaotong University, Xi’an;; 3 Guangdong Provincial Key Laboratory of Liver Disease Research, The Third Affiliated Hospital, Sun Yat-sen University, Guangzhou, P.R. China

**Keywords:** Kupffer cell, monocyte activation, liver cirrhosis, liver myofibroblasts, liver immunology

## Abstract

The ability of lymphocytes and macrophage-derived cytokines and chemokines to modulate the activation of stromal cells during immune responses is well-documented, but few studies have investigated whether liver myofibroblasts shape the phenotype and function of monocytes in liver disease. In the present study, Kupffer cells were demonstrated to be activated in the inflamed livers of patients with cirrhosis and be in close contact with liver myofibroblasts. The Kupffer cells from cirrhotic livers expressed significantly elevated levels of PD-L1 (also termed B7-H1), TLR4, CD80, CD32 and CD64 relative to those from normal livers. Consistent with this finding, the expression of these surface molecules was significantly upregulated in monocytes following exposure to liver myofibroblasts originating from inflamed livers. Accordingly, the liver myofibroblast-exposed monocytes exhibited a significant increase in dextran endocytosis. These data reveal that bidirectional interactions between liver myofibroblasts and Kupffer cells may function as an ‘amplification loop’ to enhance inflammation further in the liver. Liver myofibroblasts are central in the pathogenesis of liver diseases and should be considered as targets for the rational design of effective immune-based anti-inflammation therapies. Furthermore, it was also demonstrated that skin fibroblasts were as effective as liver myofibroblasts at inducing monocyte activation, suggesting that fibroblasts, which are numerous in the body, may represent an underrated cell population that is actively involved in immunomodulatory functions.

## Introduction

Kupffer cells, the resident hepatic macrophages, are key in hepatic fibrogenesis and are targets of proinflammatory mediators (1–3). Kupffer cells are involved in the liver fibrogenic processes via the production of cytokines and growth factors which induce the myofibroblastic transformation of hepatic stellate cells (HSCs) and also via the regulation of the production of metalloproteinases and their inhibitors (4). In response to environmental signals, Kupffer cells acquire special phenotypic characteristics with diverse functions (5). Therefore, Kupffer cell activation is best described as a wide spectrum of gradual alterations to the cell phenotype, resulting from a complex interplay of various activators and signaling pathways (6). It is well-established that the number of macrophages increases during chronic liver injury and fibrogenesis (7), although detailed phenotypic characterizations of human intrahepatic monocyte-derived cells are lacking at present.

Liver myofibroblasts (LMFs), which are principally derived from activated HSCs (8), are able to remodel the liver stroma in response to injury. Thus, LMFs, which exhibit fibrogenic and contractile properties (9), are considered to be a major fibrogenic hepatic cell type (10). Furthermore, LMFs are also involved in promoting hepatic inflammation (11). LMFs accumulate in injured hepatic areas, where they express cell adhesion molecules and secrete a number of proinflammatory factors, including interleukin (IL)-6, hepatocyte growth factor (HGF) and vascular endothelial growth factor (VEGF), to support the adhesion and migration of infiltrating lymphocytes (11). In murine models of liver fibrosis, activated HSCs express the coinhibitory molecule, B7-H4 which provides a signal to dampen antigen-specific T cell responses to modulate T cell immunity (12). Activated HSCs are also able to control the development of T cell immunity in a non-major histocompatibility complex (MHC)-restricted fashion by directly interacting with T cells in a CD54-dependent manner (13). However, at present, it is unclear how these findings from mouse models precisely relate to liver diseases in humans.

The ability of lymphocytes and macrophages to modulate the activation of stromal cells during immune responses is well documented (2,14), although little is known about the possible role of LMFs in the immune function of the human liver. In particular, few studies have investigated whether LMFs shape the phenotype and function of monocytes in liver disease. Since sinusoids have numerous open pores, LMFs are also able to interact with the sinusoid lumen, where antigen-presenting cells (APCs), including dendritic cells (DCs) and liver macrophages or Kupffer cells are present (15,16). Consistent with this property, a previous study demonstrated that coculture with HSCs differentially affected the expression of chemokine receptors and activation markers of subsets of monocytes (17). These observations led us to investigate whether LMFs secrete potent factors to regulate the phenotype and function of monocytes within inflamed human livers.

The phenotypes of Kupffer cells in cirrhotic livers were observed in the present study and the association between LMFs and Kupffer cells was investigated. Kupffer cells were shown to be activated, with a concomitant increase in the expression of certain surface molecules in fibrotic livers, while LMFs regulated the phenotype and function of monocytes through specific soluble factors. These results suggest that myofibroblasts are directly involved in regulating the function of monocytes in the human liver and that bidirectional interactions are present between the monocytes and LMFs in the liver microenvironment.

## Materials and methods

### Specimens

Human specimens were obtained from patients attending the Sun Yat-sen University affiliated hospitals following approval by the ethics committee and receiving informed patient consent. The cirrhotic liver tissues were obtained from patients undergoing transplantation for alcoholic liver disease, hepatitis B- or C-associated cirrhosis, drug-induced liver disease, Wilson’s disease or cryptogenic cirrhosis. The liver tissues from patients with hepatic hemangioma were used as the normal controls.

### Tissue immunofluorescence

For the immunofluorescence analysis, the liver tissues were cut into 5-*μ*m sections which were subsequently stained with polyclonal mouse anti-human CD68 (R&D systems, Minneapolis, MN, USA) primary antibody and rabbit anti-human α-smooth muscle actin (α-SMA) and fibroblast activation protein (FAP) primary antibodies (Abcam, Cambridge, MA, USA) followed by Alexa Fluor 488- or 568-conjugated goat anti-mouse IgG and Alexa Fluor 568- or 488-conjugated goat anti-rabbit IgG (Invitrogen, Carlsbad, CA, USA) secondary antibodies. The positive cells were detected by confocal microscopy.

### Isolation of LMFs and normal skin fibroblasts

LMFs and normal skin fibroblasts were isolated as described previously (18). The LMFs and normal skin fibroblasts were passaged for 3–8 passage doublings and were used for the subsequent experiments to minimize the clonal selection and culture stress which may occur during extended tissue culture.

### LMFs immunofluorescent staining

The LMFs were cultured on collagen-coated coverslips (Corning, Inc., Corning, NY, USA) and were fixed with 1:1 acetone/methanol for 10 min, rinsed and prewetted with phosphate-buffered saline (PBS)/10% fetal calf serum (FCS)/0.1% sodium azide. Next, the cells were stained with antibodies against fibroblast surface protein (FSP), vimentin, FAP, desmin, α-SMA, fibronectin and immunoglobulin G (IgG; Abcam) in Tris-buffered saline (pH 7.4) for 60 min. The cells were washed and incubated for 20 min in isotype-relevant goat anti-mouse fluorescein-isothiocyanate (Invitrogen) and the nuclei were counterstained with 4′,6′-diamidino-2-phenylindole hydrochloride (Sigma, St. Louis, MO, USA). The images were viewed and assessed using a fluorescence microscope (LEICA DMI 4000B) and analyzed using the Leica Application software suite (version 4.0).

### Isolation of monocytes

Peripheral blood mononuclear cells (PBMCs) were isolated from the buffy coats derived from the blood of healthy donors using Ficoll density gradients, as described previously (19). The monocytes were selected from the PBMCs using anti-CD14 magnetic beads (Miltenyi Biotec, Bergisch Gladbach, Germany) and fresh tissue monocytes were obtained as previously described (20). In brief, the liver biopsy specimens (n=12) were cut into small sections and digested in RPMI-1640 medium (Sigma) supplemented with 0.05% collagenase IV (Sigma), 0.002% DNase I (Roche Diagnostics, Mannheim, Germany) and 20% FCS (Hyclone Laboratories, Inc., Logan, UT, USA) at 37°C for 20 min. The dissociated cells were filtered through a 150-*μ*m mesh and separated by Ficoll centrifugation. The mononuclear cells were washed and resuspended in media supplemented with 1% heat-inactivated FCS for the flow cytometry analysis.

### Coculture of monocytes with LMFs or skin fibroblasts

The monocytes were cultured in DMEM (Sigma) with 10% FCS in 48-well flat-bottom microtiter plates (Corning, 2.5×10^5^ cells per well) in the presence of either LMFs or skin fibroblasts (monocyte/LMF or skin fibroblast ratio: 5/1). At the indicated time intervals, the monocytes were harvested, counted and analyzed.

### Multiplex bead-based enzyme-linked immunosorbent assay analysis of cell supernatants

Supernatants were generated by seeding 5×10^4^ cells per well into 48-well plates in 500 *μ*l phenol-red-free DMEM/1% bovine serum albumin (BSA), containing 2 mmol/l L-glutamine, 60 *μ*g/ml benzylpenicillin and 100 *μ*g/ml streptomycin (all purchased from Sigma). The Multiplex bead-based enzyme-linked immunosorbent assay analysis of the conditioned supernatants was performed using the Human 38-plex antibody bead kit, the Human 11-plex antibody bead kit and the Human 23-plex antibody bead kit (Millipore, Billerica, MA, USA) and was analyzed using a Luminex plate reader and Milliplex analyst software (Luminex 200 System).

### Flow cytometry

The peripheral blood monocytes, liver monocytes and LMFs were stained with fluorochrome-conjugated antibodies against PD-L1, PD1, CD14, CD16, CD23, CD32, CD64, CD80, CD86, TLR2, TLR4, CD166, CD90, CD29, CD73, CD13, CD44, CD105, CD31, CD45, CD34 and control antibodies (BD Biosciences, Franklin Lakes, NJ, USA or eBioscience, San Diego, CA, USA), according to the manufacturer’s instructions. The cells were subsequently analyzed using multicolor flow cytometry.

### Statistical analysis

The results are expressed as the mean ± SEM. Normality was tested using the Shapiro-Wilk test and the normally distributed data were compared using paired t-tests for associated samples, analysis of variance or independent t-tests. Non-normally distributed data were compared using the Wilcoxon signed-ranks test for associated samples or the Mann-Whitney U test for independent samples. SPSS statistical software (version 13.0) was used for all the statistical analyses. Unless otherwise specified, all the data were analyzed using two-tailed tests and P<0.05 was considered to indicate statistically significant differences.

## Results

### Kupffer cells are activated in fibrotic livers and are in contact with LMFs

**Figure 1:**
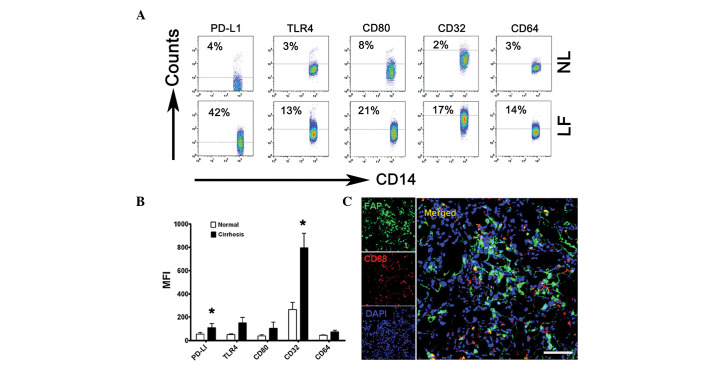
Kupffer cells were activated in the cirrhotic livers and were in contact with LMFs. (A and B) Flow cytometry analysis of PD-L1, TLR4, CD80, CD32 and CD64 expression on freshly isolated monocytes from NL (from 3 patients with hepatic hemangioma) and LF (from 12 patients with liver failure). (A) The percentage of expression of PD-L1, TLR4, CD80, CD32 and CD64 on CD14^+^ monocytes and (B) the MFI of these molecules are shown. The data in (A) are representative dot plots of ≥7 individuals from >5 independent experiments; (B) shows the statistical analysis of these samples. The results are expressed as the mean ± SEM. Significant differences in comparison with normal livers are indicated (^*^P<0.05). (C) Analysis of LMF (FAP^+^) and Kupffer cell (CD68^+^) distribution in cirrhotic liver samples by confocal microscopy. The micrographs show the contact of the LMFs and Kupffer cells; 1 out of 10 representative micrographs is shown. Bar, 200 *μ*m. LMF, liver myofirbroblast; NL, normal livers; LF cirrhotic livers; MFI, mean fluorescence intensity; FAP, fibroblast activating protein.

To identify the phenotypic features of Kupffer cells, flow cytometry was used to analyze monocytes freshly isolated from the tissues of 12 patients with cirrhosis undergoing liver transplantation and liver tissues of 3 patients with hepatic hemangioma as the normal controls. Compared with the normal controls, the monocytes isolated from the fibrotic liver samples had a significantly greater proportion of CD32^+^CD14^+^ cells (17%; Fig. 1A and B, P<0.05) and expressed significantly larger amounts of PD-L1 (42%; Fig. 1A and B, P<0.05). The results also revealed that the expression levels of CD64, CD80 and TLR4 on the monocytes were higher in the fibrotic livers than in the normal liver tissues, although the increase in the absolute mean fluorescence intensity (MFI) of the three subsets did not exhibit statistically significant differences (Fig. 1B). The differences between the phenotypes of the normal and cirrhotic liver monocytes indicate that the fibrotic environment is capable of promoting the differentiation of monocytes *in situ*.

Since Kupffer cells are critical for initiating and maintaining HSC responses, the association between Kupffer cells and LMFs in the cirrhotic livers was subsequently investigated, with particular attention to the microlocalization of the cells. Using confocal microscopy, the Kupffer cells (mainly expressing CD68) were demonstrated to be in close contact with the LMFs (highly expressing FAP), suggesting that the LMFs may regulate the function of Kupffer cells via certain signals (Fig. 1C). Collectively, these data indicate that Kupffer cells are activated in the inflamed livers of patients with cirrhosis and are in contact with LMFs.

### Phenotype of LMFs from fibrotic livers

**Figure 2:**
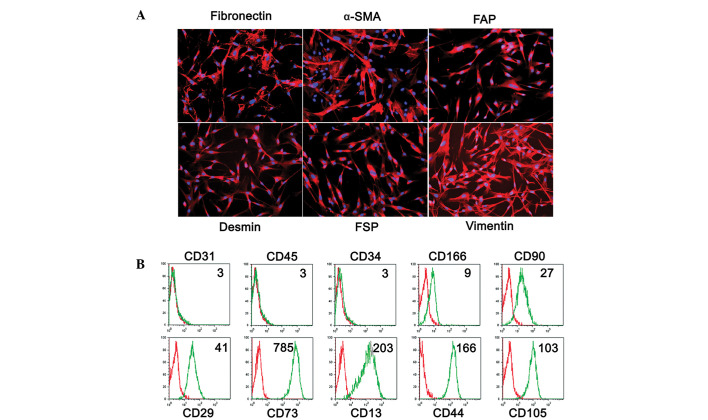
Phenotypic characterization of LMFs isolated from human liver tissues. (A) Immunofluorescent staining of LMFs isolated from a representative sample of cirrhotic livers with anti-α-SMA, fibronectin, FSP, vimentin, desmin and FAP. (B) The surface markers of the LMFs cultured for 3–5 population doublings were determined by flow cytometry. The red lines represent LMFs stained with the control antibodies (Isotype) and the green lines represent LMFs stained with the indicated antibodies (Antibody). Representative data of the MFI of LMFs are shown. The purity of the LMFs was confirmed using the endothelial, epithelial and hematopoietic markers, CD31, CD45 and CD34. The samples collected were the same as in Fig. 1. The data shown are representative of ≥7 individuals from >5 independent experiments. LMF, liver myofibroblast; α-SMA, α-smooth muscle actin; FSP, fibroblast surface protein; FAP, fibroblast activating protein; MFI, mean fluorescence intensity.

A total of 7 primary LMF cell lines were established from patients with cirrhosis and the cell phenotypes were analyzed by immunofluorescence and cytofluorimetric analyses. As shown in Fig. 2, the cultures were of high purity, with characteristic spindle-shaped cells that expressed the fibroblast markers fibronectin, α-SMA, FAP, desmin, FSP, vimentin, CD166, CD90, CD29, CD73, CD13, CD44 and CD105. Additionally, staining for CD31, CD45 and CD34 was used to exclude contamination with endothelial, epithelial and hematopoietic cells. As the expression of FAP has been reported to be a distinctive feature of activated fibroblasts (21), the phenotypic profile suggests an activated state for the LMFs. Notably, no differences were observed in the phenotype of the LMF subsets between the different underlying etiologies of cirrhosis (data not shown), suggesting that the qualitative changes in the LMF compartment represent a somewhat uniform response during fibrogenesis.

### LMFs and skin fibroblasts promote monocyte activation in vitro

**Figure 3:**
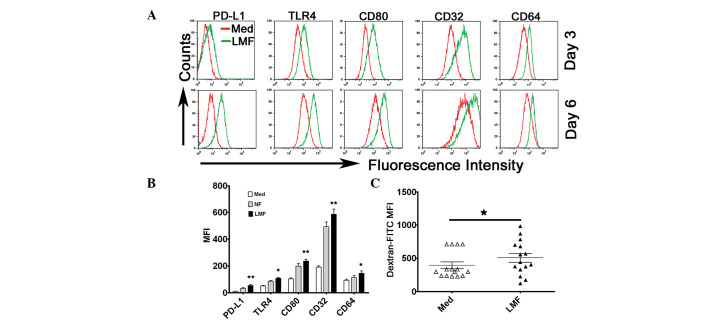
LMFs regulated PD-L1, TLR4, CD80, CD32 and CD64 expression in monocytes. (A and B) Monocytes were Med or cocultured with NF or LMFs for different time periods. The histograms are representative of 6 separate experiments. (B) Statistical analysis of the MFI with regard to the expression of the surface markers, PD-L1, TLR4, CD80, CD32 and CD64, on the monocytes following 6 days of coculture. (C) The monocytes were left untreated or pretreated for 6 days with the LMFs and were subsequently incubated for 30 min with FITC-dextran at the indicated concentrations (ng/ml). The endocytotic function of the monocytes was assessed by flow cytometry. The values in (B) and (C) represent the mean ± SEM of 6 separate experiments. ^*^P<0.05 and ^**^P< 0.01 indicate significant differences from the untreated monocytes (B and C). LMF, liver myofibroblast; Med, untreated; NF, normal skin fibroblasts.

To investigate the possible effect of LMFs on monocytes, the cells were cultured together. Monocytes, freshly isolated from the blood of unrelated healthy donors, were cocultured with various LMF cell lines for 3 or 6 days. The expression of surface molecules on the monocytes (including PD-L1, TLR4, CD80, CD32 and CD64) was subsequently analyzed by flow cytometry. The results showed that the LMFs were potent at promoting the activation of the monocytes which exhibited phenotypic features similar to the monocytes isolated from the cirrhotic livers (Fig. 3A and B). It is notable that, although these molecules were significantly upregulated in the monocytes following exposure to the LMFs for 3 days of coculture, the expression became more evident at day 6 (Fig. 3A). Similar to the LMFs, normal skin fibroblasts also affected the phenotype of monocytes and although the modulation effect of the LMFs appeared to be stronger than that of the normal skin fibroblasts, no statistically significant difference was observed (Fig. 3B). Therefore, the same effect may exist in the activation of monocytes exposed to fibroblasts of various origins.

To investigate the endocytotic ability of the monocytes following pretreatment with the LMFs further, FITC-dextran was supplied for 30 min in the coculture system. On days 3 and 6, the LMF-exposed monocytes demonstrated significant increases in dextran endocytosis (Fig. 3C). Moreover, no correlation was observed for the ability of the LMFs to augment the monocytic response and the different underlying etiologies of cirrhosis.

To obtain further insight into how the LMFs or skin fibroblasts were able to modulate the monocytic response, a method was designed to determine whether the enhancing effect was due to diffusible factors or required direct cell-to-cell interactions. After purified monocytes were cultured in conditioned medium from LMFs or skin fibroblasts, it was demonstrated that the supernatant from the fibroblasts also effectively induced the activation of monocytes, suggesting that certain soluble factors were secreted to modulate the monocytes (data not shown).

Taken together, these results suggest that LMFs are critical in maintaining the activation of monocytes in the fibrotic liver environments of humans.

### LMFs and skin fibroblasts may regulate the phenotype and function of monocytes through types of cytokines, chemokines and growth factors

**Figure 4:**
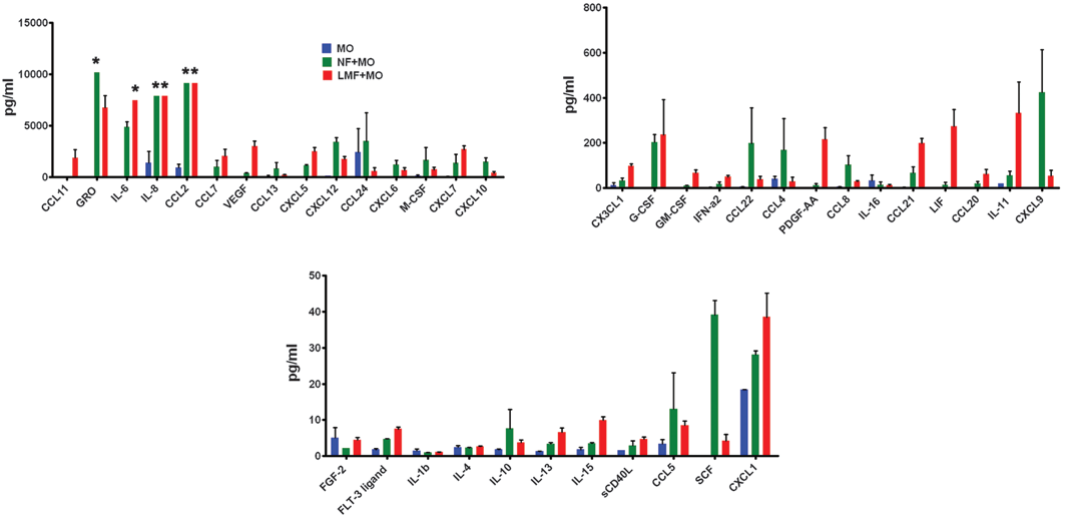
Interaction of monocytes with LMFs or skin fibroblasts caused a rise in the levels of various cytokines, chemokines and growth factors. The levels of various factors in the cell-free culture supernatants of the MO and the coculture systems of NF+MO or LMF+MO were assessed with a Multiplex bead-based enzyme-linked immunosorbent assay at day 6. The data are expressed as the mean ± SEM of triplicates. Asterisks indicate levels beyond the detectable range. LMF, liver myofibroblast; MO monocytes; NF+MO, monocytes with normal skin fibroblasts; LMF+MO, liver myofibroblasts with normal skin fibroblasts.

The aforementioned observations indicate that the LMFs may supply locally acting paracrine cues which induce monocyte activation within the fibrotic liver environment. To understand this crosstalk more thoroughly, *in vitro* cocultures of monocytes and LMFs/skin fibroblasts were established and their conditioned media were screened for levels of various cytokines, chemokines and growth factors using the Multiplex bead-based enzyme-linked immunosorbent assay. Notably, the levels of the majority of the cytokines, chemokines and growth factors in the cocultures were higher than those produced by the monocytes alone (Fig. 4).

## Discussion

Over the past decade, considerable research has been focused on Kupffer cell-mediated liver injury. Kupffer cells are the best-characterized targets of lipopolysaccharide (LPS) in the liver (22,23) where they are crucial in hepatic fibrogenesis through the enhancement of HSC activation (24,25). However, little is known concerning whether LMFs affect the differentiation and function of Kupffer cells. The present study demonstrates that the LMFs from cirrhotic livers modulate the phenotype and function of monocytes which may represent a novel link between inflammation and fibrosis in the liver.

The liver consists of hepatic parenchyma and a large proportion of nonparenchymal cells (NPCs), including sinusoidal endothelial cells, Ito cells and dedicated hepatic macrophages (Kupffer cells) (26). Kupffer cells are important in the normal physiology and homeostasis of the liver and participate in the acute and chronic responses to toxic compounds. The direct or indirect activation of Kupffer cells by toxic agents results in the release of an array of inflammatory mediators, growth factors and reactive oxygen species and this activation appears to modulate hepatocyte injury. In the present study, the Kupffer cells in diseased livers were observed to exhibit activated phenotypes with increased expression of PD-L1, CD80, CD32, CD64 and TLR4 (Fig. 1). Notably, these activated Kupffer cells were in close contact with LMFs, suggesting that such monocytes may actually be modulated by LMFs. This theory is supported by the subsequent finding that the phenotype and function of monocytes were correlated with the LMFs in coculture (Fig. 3).

LMFs originate principally from activated HSCs. However, in fibrotic disease, subpopulations arise from other sources, such as bone-marrow precursors (27–29). Since it is hypothesized that myofibroblasts isolated from tissues express imprinted phenotypes that are stable in culture (30), the behavior of these cells *in vitro* is likely to reflect their function *in vivo*(31). Differentiated LMFs isolated directly from diseased human livers were studied. The isolated myofibroblasts were positive for fibronectin, α-SMA, FAP, desmin, FSP, vimentin, CD166, CD90, CD29, CD73, CD13, CD44 and CD105, whereas the characteristic markers of epithelial, endothelial or hematopoietic cells, including CD31, CD45 and CD34, were negative. There were no consistent differences that characterized the LMFs isolated from the various diseased livers and all the LMFs expressed the same types of markers (Fig. 2). Consistent with the results of the present study, other investigators have reported that LMF preparations from various diseased livers expressed similar patterns of proinflammatory cytokines and chemokines (11).

Monocytes are versatile, plastic cells that respond to environmental signals through diverse functional programs (32,33). Other investigators have demonstrated that LMFs secrete potent lymphocyte chemotactic factors when stimulated by proinflammatory cytokines. The present study provides evidence that certain cytokines, chemokines and growth factors exist in LMFs and skin fibroblast coculture systems with monocytes. These soluble factors may promote monocytes activation. Therefore, LMFs may represent a novel mechanism which modulates monocyte immunity. Moreover, further detail was provided on these soluble factors using the Multiplex bead-based enzyme-linked immunosorbent assay and the results of the present study are likely to aid future studies.

The skin fibroblasts were as effective as the LMFs at inducing the activation of monocytes, suggesting that fibroblasts, which are numerous in the body, may represent an underrated cell population that is actively involved in immunomodulatory functions. However, the mechanisms involved in the activation of monocytes may differ between the LMFs and skin fibroblasts for a number of the distinct soluble factors in the coculture systems.

The observation that LMFs modulate the phenotype and function of Kupffer cells provides a mechanism whereby LMFs may determine the immune status within the inflamed liver. There is a fine-tuned collaborative interaction between immune cells and LMFs in liver microenvironments. Indeed, bidirectional interactions between LMFs and Kupffer cells may function as an ‘amplification loop’ to enhance inflammation further in the liver, thereby extending the role of the LMFs in liver disease from fibrogenesis to an active role in regulating inflammation. Although these regulatory loops must be studied in more detail, the present study provides novel insights into the mechanisms underlying hepatic inflammation and particularly the role of LMFs as proinflammatory elements. Together with previous studies indicating that HSCs act as antigen-presenting cells (34), the findings of the present study suggest that LMFs are central to the pathogenesis of liver disease and may be important therapeutic targets for reversing liver inflammation.
